# Society Proceedings

**Published:** 1895-08

**Authors:** 


### SOCIETY PROCEEDINGS.

### NEW YORK STATE VETERINARY MEDICAL SOCIETY.

THE sixth annual meeting will be held at the lecture-rooms of the New York College of Veterinary Surgeons, No. 154 East Fifty-seventh Street, New York City, on September 5 and 6, 1895.

The programme for the two days' proceedings will be well filled with interesting and instructive events. There will be the election of officers, the President's annual address, the reports of the Secretary-Treasurer and of the several standing committees, including that of the Committee on Legislation, which has done such glorious work in the past year by securing the passage of the bill regulating the practice of veterinary medicine and surgery in this State, placing this branch of the science on the same footing with our medical brethren. There will also be reports from the several County Secretaries.

Several members of the Society have signified their willingness to prepare essays on subjects of importance, which they will read and defend. The Committee of Arrangements have secured ample hotel accommodations for all attending members close by the place of meeting. The New York County Society has also made arrangements to entertain the members of the State Society while in their city. The Committee of Arrangements has also under consideration the feasibility of setting apart a half-day of our meeting for the purpose of having a practical demonstration, in the way of having several operations on live subjects, such as arytenectomy, neurectomy, and Cunean tenotomy, and any other operation that will be interesting and instructive to the members. The officers of the Society are making every endeavor to make this the most successful, instructive, interesting, and best-attended meeting that has ever been held.

Besides the above-mentioned events of this meeting there will be other important and interesting proceedings to bring before the members of this Society for them to discuss and act upon, such as the changing of the by-laws, the explanation of the law recently passed regulating the practice of veterinary medicine in this State, the publishing of a New York State Veterinary Register, the election of delegates for the United States Veterinary Medical Association meeting, and several other matters of importance and interest to every veterinarian in this State, will be taken up, discussed and acted upon.

It is expected that there will not be a dull hour on the programme of the meeting. All members and all qualified veterinarians are cordially invited to attend. Printed notices, with programme, will be sent to every qualified veterinarian in this State. Blank application for membership can be had by addressing the Secretary, No. 395 Ellicott Street, Buffalo, N. Y.

NELSON P. HINKLEY,
Secretary.

### MISSOURI VALLEY VETERINARY ASSOCIATION.

THE first annual meeting was held in the parlors of the National Hotel, Leavenworth, Kansas, and was called to order promptly at 7.30 P.M. by President Stewart. Drs. Barth, Bray, Ernst, Harrison, Hunter, Sihler, Stewart, Wattles, G. T. Netherton, and Biart, responded to roll-call. Others present were Drs. Airth, Topping, Thompson, Hill, C. O. Netherton, and Mr. E. J. Netherton, a student of the Kansas City Veterinary College; also Dr. McGill, of Leavenworth. Letters of regret were read from Drs. Bell, Pritchard, Mayo, Moore, Meyers, Day, Turner, Klutts, and others.

The annual report of the Secretary-Treasurer was read and approved, and ordered filed. A vote of thanks was given the Secretary for the manner in which he had performed his duties.

The Board of Censors reported favorably upon the following petitions, and the gentlemen were duly elected to membership: Drs. Airth, Thompson, Hill, Topping, C. O. Netherton, King, Kaupp, Cock, and Young.

The following resolution was unanimously adopted: *Resolved*, That it is the sense of this Association that no contagious pleuro-pneumonia exists or has ever existed in Kansas.

Dr. Barth, of Kansas City, read an interesting paper on "Actinomycosis."[1] In discussion, Dr. Bray asked if range-cattle, fed on nothing but grass, are affected. Dr. Barth said that they were. Dr. Harrison asked if discharge falling on grass would carry infection. Dr. Barth said, yes. Dr. Stewart said that the germs of actinomycosis are found on grass and grain; has seen young cattle, fed on stalks in winter and grass in summer, with the disease. Dr. Netherton asked if age had any bearing as a cause of the disease. Dr. Barth replied that he had seen cases well advanced in years. Dr. Netherton considered it more liable to attack young cattle; thought perhaps teething would facilitate the introduction of the virus. Dr. Sihler had seen cases in old cows and bulls, and apparently new cases; but such animals are generally sold before the disease becomes well advanced. Dr. Netherton asked if, when aged cattle are affected, the teeth are sound. Dr. Wattles stated that as the disease is caused by spores, it can attack any animal having an abrasion of the mucous membrane, and inquired if any had tried the iodide-of-potash treatment, and what results. Dr. Harrison had given one drachm of iodide of potash each day until ten doses had been given; had also used a saturated solution of the same, and injected the tumor once. The cattle treated were sold and brought the market price. Dr. Sihler had had no personal experience, but had advised farmers to try it, and one farmer had reported three steers cured in two weeks' treatment. Dr. Ernst had used the treatment, and it was effectual. Dr. Stewart gave results of experiments at Omaha, confirming the iodide-of-potassium treatment. Dr. Wattles said he could understand how it will cure cases where the glands alone are diseased, but thought when the bone is diseased the treatment would do no good. Dr. Barth said that cases of slight bone-affection may be cured.

Dr. Sihler's paper on "Fever" was a most excellent one and well discussed.

[1] See page 487.

Dr. Ernst's paper, "A Selection in Cattle as a Preventive of Disease Among People,"[1] was good and well received. It was discussed by Drs. Stewart, Harrison, and Netherton.

Dr. Harrison read a paper on an outbreak of "Epizoötic Abortion"[2] among a herd of cows in Australia. Discussed by Drs. Wattles, Biart, Stewart, and Bray. Injections of permanganate of potassium were used by some, others used a solution of boric acid, and others preferring corrosive sublimate, 1: 2000, and cleansing of all the parts, external as well as internal. The administration of viburnum prunifolium was recommended, also isolation and thorough disinfection. Dr. McGill referred to the occurrence of the disease among humans, and also an attack he had witnessed among cattle. Dr. Airth reported cases, and considered isolation and disinfection for two or three weeks a necessity, Dr. Airth laid special stress on isolation, as the disease would go through the whole herd if this was neglected. Dr. Wattles would irrigate the uterus as well as the vagina. Dr. Biart thought disinfection does no good; believes in separation, spaying, and fattening. Dr. Stewart thought irrigation is not always essential. Dr. Wattles thought irrigation not always necessary, especially where isolated cases occur. Dr. Airth coincided with Dr. Wattles.

Dr. Stewart exhibited some cuts made of the different stages of growth of *echinococcus veterinorum*, including mother-and-daughter cysts, mounting specimens and showing the parasite most beautifully. He also explained its life-history. He said that the dog and the hog were most liable to attack, the dog showing symptoms very similar to rabies. Dr. Stewart was assisted by Dr. Wattles. A vote of thanks was given the gentlemen.

The following resolution was unanimously adopted: *Resolved*, That this Association is in favor of the appointment of none but graduates in veterinary medicine to the positions of city meat and milk inspectors, and also in favor of having at least one veterinarian as a member of each board of public health.

The election of officers resulted as follows: Dr. Sesco Stewart, *President;* Dr. R. H. Harrison, *First Vice-President;* Dr. G. T. Netherton, *Second Vice-President;* Dr. S. L. Hunter, *Secretary-Treasurer;* Drs. Bray, Hill, Wattles, Sihler, and Barth, *Board of Censors.*

The meeting then adjourned to meet at Kansas City, Mo., in September, subject to the call of the President.

S. L. HUNTER, V.S.,
Secretary-Treasurer.

---

### VIRGINIA STATE VETERINARY MEDICAL ASSOCIATION.

THIS Association met in the Veterinary Department of the Virginia Agricultural and Mechanical College at Blacksburg, on Tuesday, June 18, 1895, in regular session. Dr. W. H. Harbaugh, of Richmond, President of the Association, delivered his annual address, after which subjects pertaining to

[1] See page 490. [2] See page 469.

the status of veterinary colleges and to veterinary education generally were discussed at length. It was learned during the discussion that a certain veterinary college had issued a diploma upon an attendance in college from last November to April. This man is now advertising extensively in this State, and competing *as a graduate* with men who have earned their diplomas. Much indignation was expressed because an institution of pretended merit should send into this State, where none are protected by any laws, men who are in reality not sufficiently educated, and whose diplomas in no way represent true qualification.

The report from the Committee on Collective Statistics was a valuable paper upon the diseases of the State. Tuberculosis, Southern cattle-fever, influenza, strangles, and pneumonia prevailed in about the order named; epizoötic pharyngitis prevailed in Richmond and Norfolk, and an outbreak of anthrax was reported from the Western part of the State.

Among the interesting cases cited, Dr. J. E. Miller reported "Rupture of the Stomach in a Stallion;" Dr. A. Bannister, of Roanoke, the "Post-mortem Lesions in a Case of Adenoma;" Dr. Niles, of Blacksburg, "A Case of Anthrax;" Dr. Faville, a case of "Pyæmia Following Pharyngitis;" Drs. Marshall and Dixon, some interesting surgical cases.

Dr. Harbaugh read a very interesting paper on "Plural Pregnancy," involving some fine points in jurisprudence.

After being most splendidly entertained by Professor Niles and his estimable wife, the Association adjourned to meet in Richmond, January 27, 1896, at which time the election of officers will take place.

GEORGE C. FAVILLE, D.V.M.,
Secretary.

